# Chemovirotherapeutic Treatment Using Camptothecin Enhances Oncolytic Measles Virus-Mediated Killing of Breast Cancer Cells

**DOI:** 10.1038/s41598-019-43047-3

**Published:** 2019-05-01

**Authors:** Chen-Jei Tai, Ching-Hsuan Liu, Yu-Chi Pan, Shu Hui Wong, Cheng-Jeng Tai, Christopher D. Richardson, Liang-Tzung Lin

**Affiliations:** 1 Department of Traditional Chinese Medicine, Taipei Medical University Hospital, Taipei, Taiwan; 2 Department of Obstetrics and Gynecology, School of Medicine, College of Medicine, Taipei Medical University, Taipei, Taiwan; 3 Graduate Institute of Medical Sciences, College of Medicine, Taipei Medical University, Taipei, Taiwan; 4 Department of Microbiology and Immunology, Dalhousie University, Halifax, Nova Scotia Canada; 5 Department of Microbiology and Immunology, School of Medicine, College of Medicine, Taipei Medical University, Taipei, Taiwan; 6 International Master Program in Medicine, College of Medicine, Taipei Medical University, Taipei, Taiwan; 7 Division of Hematology and Oncology, Department of Internal Medicine, Taipei Medical University Hospital, Taipei, Taiwan; 8 Department of Internal Medicine, School of Medicine, College of Medicine, Taipei Medical University, Taipei, Taiwan; 9 Department of Pediatrics and Canadian Center for Vaccinology, Izaak Walton Killam Health Centre, Halifax, Nova Scotia Canada

**Keywords:** Measles virus, Viral vectors, Breast cancer, Molecular medicine

## Abstract

Oncolytic virotherapy represents an emerging development in anticancer therapy. Although it has been tested against a variety of cancers, including breast cancer, the efficacy of oncolytic viral vectors delivered as a monotherapy is limited. Enhancing viral oncolytic therapies through combination treatment with anticancer agents is a feasible strategy. In this study, we considered a chemovirotherapeutic approach for treating breast adenocarcinoma using oncolytic measles virus (MV) and the chemotherapeutic agent camptothecin (CPT). Our results demonstrated that co-treatment of MV with CPT yielded enhanced cytotoxicity against breast cancer cells. Low dosage CPT combined with MV was also found to elicit the same therapeutic effect as high doses of CPT. At the lower dosage used, CPT did not inhibit the early stages of MV entry, nor reduce viral replication. Further studies revealed that co-treatment induced significantly enhanced apoptosis of the breast cancer cells compared to either MV or CPT alone. Overall, our findings demonstrate the potential value of MV plus CPT as a novel chemovirotherapeutic treatment against breast cancer and as a strategy to enhance MV oncolytic activity.

## Introduction

As one of the most commonly known adenocarcinomas in the world^1^, breast cancer remains a major cause of death in women despite improved survival rates with advances in hormone therapy, chemotherapy, surgery, and radiotherapy^2^. While prognosis tends to be favorable for early-stage breast cancers, metastatic breast cancers are usually very difficult to treat and are often incurable^3^. Continued research into novel and more effective therapies is critical.

Oncolytic virotherapy is a promising treatment that selectively targets and destroys cancer tissues with minimal damage to normal cells^4–7^. Most cancer therapies are non-specific to tumor cells, while oncolytic virotherapy capitalizes on properties of the transformed cells that favor the replication of specific viruses^3^. Wild-type, attenuated, and genetically modified viruses target a diverse collection of cancers, including breast adenocarcinoma. The recent discovery that the tumor marker Nectin-4 (also known as poliovirus receptor related protein 4; PVRL4), which is frequently overexpressed in many adenocarcinomas^8–11^, can function as a receptor for measles virus (MV)^12,13^, has suggested the use of this virus for treating breast cancers^14^.

MV is an enveloped, negative-sense, single-strand RNA virus. Presently, three cellular receptors have been identified for MV and include membrane cofactor protein (CD46), signaling lymphocytic activation molecule (SLAM; also known as CD150), and the adherens junction protein Nectin4 (PVRL4)^14^. CD46 and PVRL4 are often upregulated in a variety of human tumor cells^8–11^. However, CD46 is also ubiquitously expressed on most normal human cells, albeit at lower levels, and is only utilized by laboratory adapted/vaccine strains MV. Nectin-4 is typically overexpressed on the surface of adenocarcinoma cells, and is utilized by both laboratory/vaccine and wild-type strains MV^14^. Consequently, wild-type MV strains that target Nectin-4 can be harnessed to selectively destroy Nectin-4-positive tumors, including those associated with breast cancer^15^.

Engineered oncolytic MV has been suggested as a potential treatment against many cancers^15,16^. Many clinical trials are under way for testing MV oncolytic activity against ovarian, fallopian, or peritoneal cancer (NCT02068794, NCT02364713, NCT00408590), malignant mesothelioma (NCT01503177), metastatic head and neck cancer, breast cancer (NCT01846091), multiple myeloma (NCT00450814, NCT02192775), and malignant peripheral nerve sheath tumor and neurofibromatosis (NCT02700230). As is the case with other oncolytics viruses, a significant challenge remains with their limited efficacy as a monotherapeutic agents^17^. A combination of oncolytic therapy with chemotherapy, known as chemovirotherapy, is a feasible approach for maximizing the anticancer potency of virotherapeutics^18^.

Camptothecin (CPT), derived from the bark of *Camptotheca acuminata*, is a cytotoxic quinoline alkaloid that exerts antitumoral activity via its inhibition on DNA topoisomerase I (Topo I)^19^. CPT type of drugs, including its analogues, the FDA-approved chemotherapeutic agents irinotecan and topotecan^20^, are known to induce apoptosis in different cancer cells^21^. MV on the other hand, produces cytopathic effects including multi-nucleated giant cell (‘syncytia’) formation and apoptosis^22–24^. A combination of oncolytic MV and CPT therapies is likely to be synergistic, since they both trigger cell death through distinct pathways. Together they may promote better killing of tumor cells, with less likelihood of the cancer developing resistance to the individual agents^25^. In order to examine the proof-of-principle of this approach, we evaluated the effects of recombinant wild-type MV together with CPT on the growth and viability of breast cancer cells. We investigated the optimal treatment model of their combined actions *in vitro*, the underlying mechanism, and demonstrated the potential of this approach for further development and treatment strategy of breast cancer.

## Materials and Methods

### Cell culture, virus, and reagents

Human breast adenocarcinoma MCF-7 (kindly provided by Dr. Ming-Sound Tsao; University of Toronto, Toronto, Canada) and T-47D (kindly provided by Dr. David W. Hoskin; Dalhousie University, Halifax, Canada) cells were cultured in Dulbecco’s Modified Eagle’s medium (DMEM; GIBCO-Invitrogen, Carlsbad, CA, USA) supplemented with 10% fetal bovine serum (FBS; GIBCO-Invitrogen), 100 U/ml of penicillin G, 100 µg/ml of streptomycin, and 0.25 µg/ml of amphotericin B (GIBCO-Invitrogen). The cells were maintained at 37 °C in a 5% CO_2_-humidified incubator. MV-EGFP (recombinant Ichinose-B 323 wild-type measles virus isolate, IC323) with enhanced green fluorescent protein tag was disseminated in marmoset B lymphoblastoid cells (B95a) as previously described^12^. Viral titers were determined by the 50% tissue culture infective dose (TCID_50_) assay, and virus concentration was indicated as multiplicity of infection (MOI). Basal medium containing 2% FBS with antibiotics was used in all viral assays and experiments. CPT (Sigma-Aldrich; St. Louis, MO, USA) and punicalagin^26^ (PUG; kindly provided by Dr. Ta-Chen Lin, Central Taiwan University of Sciences and Technology, Taiwan) were dissolved in DMSO prior to use. The final DMSO concentration in the drug solutions was equal to or below 0.1%.

### Cell viability assay

Cells seeded in 96-well plates (1 × 10^4^ cells per well) were treated with a range of CPT concentrations or MV-EGFP at varying MOI for 72 or 120 h. Cell viability was assessed using the MTT (3-(4,5-Dimethyl-2-thiazolyl)-2,5-diphenyl-2H-tetrazolium bromide) colorimetric assay (Millipore; Billerica, MA, USA). MTT was prepared in serum-free medium at 0.5 mg/ml final concentration and added to the cells at 100 µl/well before incubation at 37 °C in 5% CO_2_ for 3 h. The supernatant was subsequently discarded and 100 µl DMSO was added per well, and the plate was shaken for 10 min. Cell viability was then evaluated by measuring the optical density (OD) at 550 nm using a microtiter plate reader. The percent cell viability was determined compared to the Mock control group and the 50% cytotoxic concentration (CC_50_) of CPT was analyzed as previously described^27^.

### Synergistic effect of MV plus CPT treatment against breast cancer cells

Cells seeded in 96-well plates (10^4^ cells per well) were studied in three different experiments of MV plus CPT administration. The first tested for ‘drug sensitization’ with the addition of CPT to cells at different concentrations (10, 30, and 50 nM) for 2 days, prior to the infection of cells with MV at a MOI 0.1 for 3 days. The second experiment consisted of a ‘viral sensitization’ treatment, where cells were first infected with MV at MOI 0.1 for 2 days, followed by CPT treatment at varying concentrations (10, 30, and 50 nM) for 3 days. Finally, the third experiment consisted of a ‘co-treatment’ group, where the cells were infected with MV at MOI 0.1 and at the same time, treated with varying concentrations of CPT (10, 30, and 50 nM) for a total of 5 days. For all experiments, MV infection was performed for 1.5 h at 37 °C and cells were washed with phosphate buffered saline (PBS) before and after viral challenge. Efficacies of the different modes of treatment were then evaluated by determining cell survival with the MTT assay as described above in the ‘Cell viability assay’. Combination index (CI) values were calculated using the Chou-Talalay method^28^ to quantitatively deduce synergistic, additive or antagonistic effects of MV plus CPT.

### Effect of CPT on free oncolytic MV particles

The assay is performed as reported elsewhere^29^ with some modifications. Cell-free virus particles were incubated with CPT at different concentrations at 37 °C in 5% CO_2_ for 3 h. The virus-drug mixture was then diluted 20-fold with 2% FBS DMEM to effective concentrations of CPT, after which the inoculum was added to MCF-7 cells seeded in 96-well plates (10^4^ cells per well; final MOI 0.1). The cells were incubated at 37 °C in 5% CO_2_ for 3 days. After 3 days, the plates were scanned and analyzed for viral reporter fluorescence using the Typhoon 9410 variable mode imager (Amersham Biosciences; Baie d’Urfe, QC, Canada) to evaluate MV-EGFP infections. Fluorescence intensity was quantified using Image Quant TL software (Amersham Biosciences).

### Effect of CPT on MV attachment

The assay is performed as described before^29^ with some modifications. MCF-7 cells were co-treated with MV (MOI 0.1) and varying concentrations of CPT (10, 30, and 50 nM) at 4 °C for 1.5 h. Subsequently, the virus-drug inocula were removed, and the cells were washed with PBS. Fresh culture medium was added to the cells, which were then incubated at 37 °C for 3 days. EGFP fluorescence intensities were measured as described above.

### Effect of CPT on MV penetration

The experiment was performed as previously reported^29^ with some modifications. MCF-7 cells were infected with MV (MOI 0.1) and incubated at 4 °C for 1.5 h. Following which, viral inocula were removed and cells were treated with varying concentrations of CPT (10, 30, and 50 nM) and shifting the incubation to 37 °C for 1.5 h. CPT was subsequently removed and the cells were washed with PBS before adding fresh culture medium. After further incubation at 37 °C for 3 days, viral EGFP signal intensities were detected and analyzed as previously described.

### Time-of-drug-addition assays

Pre-treatment, co-addition, and post-infection CPT drug treatments were performed as previously described^27^ to determine potential antiviral effect of CPT on oncolytic MV infection at different time-points. For the pre-treatment group, MCF-7 cells seeded in 96-well plates (10^4^ cells per well) were treated with CPT (10, 30, and 50 nM) for 24 h and washed before infection with MV (MOI 0.1) for 48 h. For the co-addition treatment group, MCF-7 cells seeded in 96-well plates (10^4^ cells per well) were treated with virus-drug inocula containing both MV (MOI 0.1) and varying concentrations of CPT (10, 30, and 50 nM) simultaneously for 1.5 h before the wells were washed and refreshed with complete culture medium for 3 days of incubation. For the post-infection treatment group, MCF-7 cells seeded in 96-well plates (10^4^ cells per well) were first infected with MV (MOI 0.1) for 1.5 h, washed, and then immediately treated with varying concentrations of CPT (10, 30, and 50 nM) for 3 days. For all three treatment groups at the end-point of the experiments (72 h), the viral reporter fluorescence was measured using the Typhoon 9410 variable mode imager and the data obtained was analyzed using Image Quant TL software. For readouts based on viral titer in the above time-of-drug-addition experiments, MCF-7 cells were seeded in 12-well plates (2 × 10^5^ cells per well) and were similarly treated with CPT (10, 30, and 50 nM) as above, either 24 h prior to, concurrently to (0 h), or right after (1.5 h) the MV infection (MOI 0.1); wash steps were included as described earlier. Supernatant was harvested from each well at 3 days post-infection for determination of viral titer by TCID_50_ analysis.

### Cell cycle analysis

MCF-7 cells seeded in 6-well plates (3 × 10^5^ cells per well) were grown overnight, before being subjected to co-treatment with MV (MOI 0.1) and varying concentrations of CPT (10, 30, and 50 nM) at 37 °C for 1.5 h. Subsequently, the virus-drug inocula were discarded and replaced with fresh culture medium containing the respective concentrations of CPT for 5 days incubation at 37 °C. At the end of the experiment, the cells were collected by trypsinization and transferred into 15 ml tubes, where they were washed with ice-cold PBS and then fixed overnight in 70% ethanol at 4 °C. Following fixation, the cells were washed twice with PBS and incubated at 37 °C for 30 min in PBS solution containing 10 mg/ml ribonuclease A from bovine pancreas (RNase A; Sigma-Aldrich). Propidium iodide (PI; Sigma-Aldrich; 40 µg/ml) was then added to the cells before incubation at 37 °C in the dark for 15 min, and cell cycle analysis was performed using the Beckman coulter FC500 flow cytometer (Beckman Coulter Inc.; Brea, CA, USA).

### Apoptosis analysis using propidium iodide and Annexin V staining

For apoptosis analysis using PI and Annexin V, MCF-7 cells were seeded in 6-well plates (3 × 10^5^ cells per well) and co-treated with MV (MOI 0.1) and CPT (10, 30, and 50 nM). At the end of the 5 days incubation period, the cells were collected by trypsinization, transferred into 15 ml tubes, and washed with ice-cold PBS. After washing, the cells were resuspended in binding buffer containing 1 µl/ml PI and 1 µl/ml Annexin V APC-conjugated (Enzo Life Sciences, Inc; East Farmingdale, NY, USA). Apoptosis analysis, which was based upon the cell surface exposure of phosphatidyl serine that binds to Annexin V, was performed using the Beckman coulter FC500 flow cytometer (Beckman Coulter Inc.).

### Western immunoblot analysis

MCF-7 cells seeded in 6-well plates (3 × 10^5^ per well) or 6 cm dishes (8 × 10^5^ cells) were grown overnight, before being subjected to co-treatment with MV (MOI 0.1) and varying concentrations of CPT (10, 30, and 50 nM) at 37 °C for 1.5 h. Following the treatment, the virus-drug inocula were discarded and replaced with fresh complete culture medium containing the respective concentrations of CPT for 5 days incubation at 37 °C. At the end of the experiment, the MCF-7 cells were harvested and lysed using RIPA buffer containing protease inhibitors (Roche Molecular Biochemicals; Indianapolis, IN, USA). Protein sample concentrations were then determined using the bicinchoninic acid (BCA) protein assay kit (Thermo Fisher Scientific Inc.; San Jose, CA, USA). Proteins were resolved by subjecting the lysates to 10% sodium dodecyl sulfate-polyacrylamide gel electrophoresis (SDS-PAGE), followed by transfer onto polyvinylidene difluoride (PVDF) membrane for western immunoblot analysis. The membrane was first blocked with 5% non-fat milk prepared in Tris-buffered saline with 0.1% Tween® 20 (TBST), before probing target proteins with primary antibodies at appropriate dilutions: poly (ADP-ribose) polymerase (PARP) antibody (1:1000; Cell Signaling Technology, Inc., Danvers, MA, USA) and β-actin antibody (1:10000; Cell Signaling Technology, Inc.). Antibody binding was detected by incubating the blot with horseradish peroxidase (HRP)-conjugated secondary antibody (GIBCO-Invitrogen) followed by treatment with the HRP chemiluminescent substrate (Millipore). Chemiluminescence was measured using the UVP BioSpectrum 500 imaging system (UVP; Upland, CA, USA). Protein signals were quantified and compared against the β-actin loading control using densitometry analysis.

### Statistical analysis

All data are expressed as means ± standard error of the means (SEM). Statistical significance was determined using one-way analysis of variance (ANOVA) followed by Dunnett’s multiple comparison. *P* < 0.05 was considered statistically significant. All assays were conducted with at least three independent experiments.

## Results

### Efficacy of cell killing of MCF-7 breast cancer cells by oncolytic MV and CPT as separate agents

**Figure 1:**
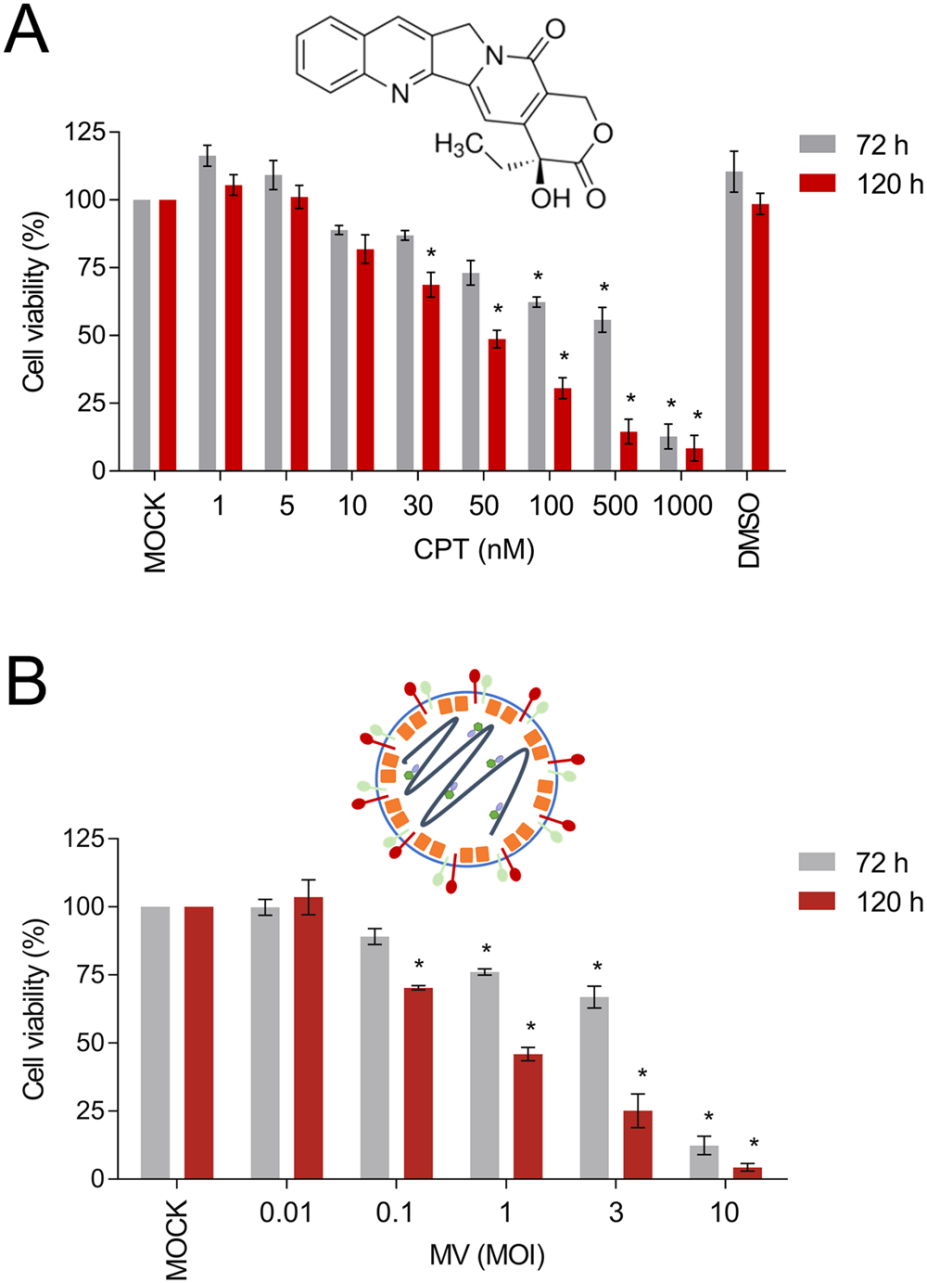
CPT and oncolytic MV are cytotoxic against human MCF-7 breast cancer cells. MCF-7 cells were treated with (**A**) CPT (1−1000 nM) or (**B**) MV (MOI 0.01, 0.1, 1, 3, and 10) for 3 or 5 days. Cell viability was analyzed by MTT assay. DMSO = 0.1%. All data shown are means ± SEM (*P < 0.05 compared to Mock treatment) from three independent experiments.

We first evaluated the ability of CPT and oncolytic MV to kill breast cancer cells as separate agents. For this purpose, we used various concentrations of CPT or different MOIs of a recombinant wild-type-based MV containing and EGFP reporter gene to treat and infect MCF-7 breast adenocarcinoma cells. The MCF-7 cells express Nectin-4/PVRL4 over their cell surfaces and are highly susceptible to MV infection^12^. Both CPT (Fig. 1A) and oncolytic MV (Fig. 1B) decreased cell viability of MCF-7 in a dose-dependent manner after either 3 or 5 days of treatment. At concentrations > 50 nM, CPT could reduce cell viability by over 50% and its CC_50_ values were determined to be 381 nM and 58.5 nM for 3 and 5 days of treatment, respectively (Fig. 1A). On the other hand, infection with oncolytic MV at a MOI > 0.1 could reduce MCF-7 cell viability by 50% or more (Fig. 1B). Similar results were obtained using lactate dehydrogenase (LDH) release cytotoxicity detection assay (Supplementary Fig. S1). Based on these results, concentrations of CPT (10, 30, and 50 nM) and oncolytic MV (MOI 0.1) were chosen for subsequent experiments designed to determine their synergistic effects in combination treatments.

### Combination treatments with oncolytic MV and CPT exert enhanced killing of human MCF-7 breast cancer cells

**Figure 2:**
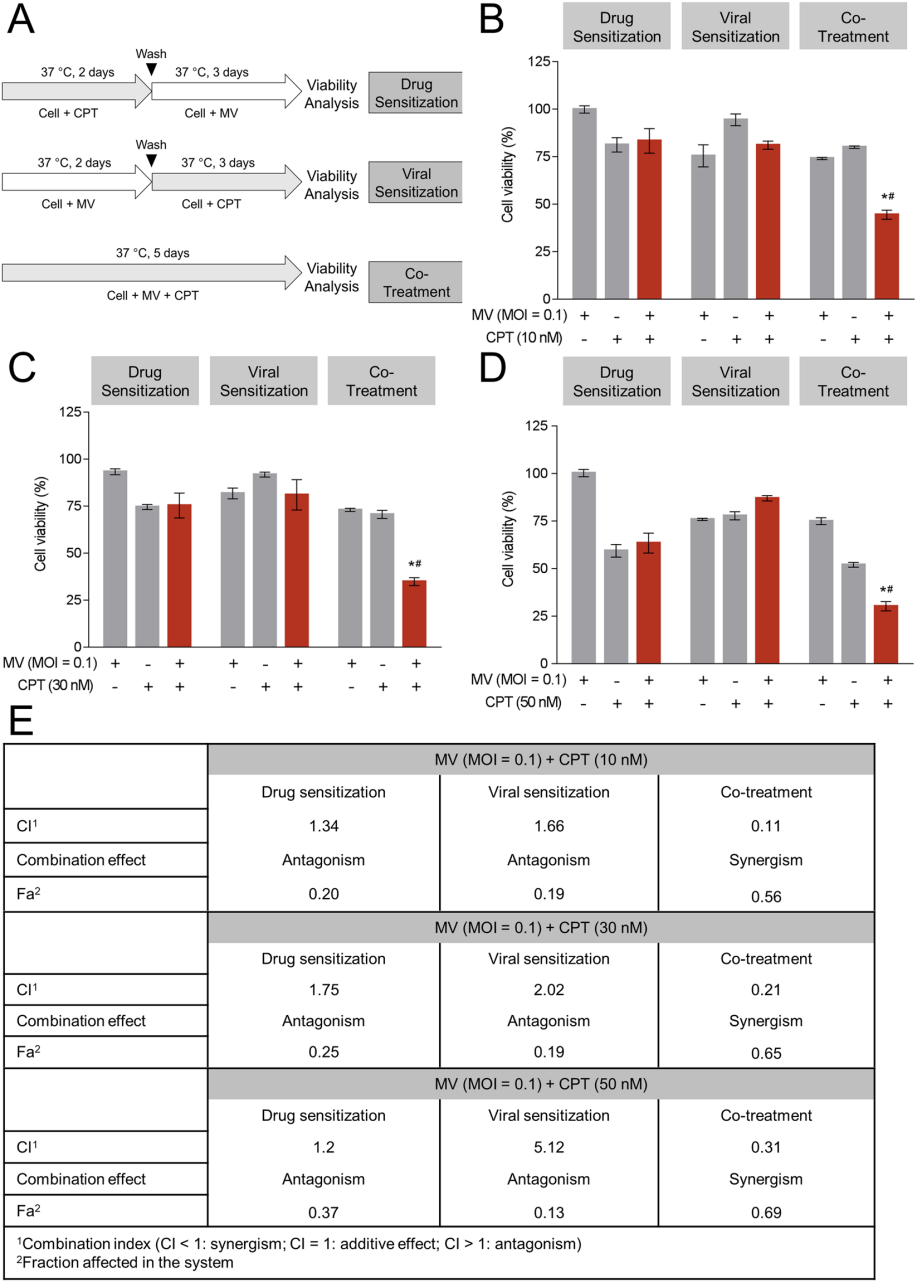
Co-treatment of MV plus CPT exhibits enhanced anticancer activity against human MCF-7 breast cancer cells. (**A**) Schematic representations of the different treatment models. (**B**–**D**) MCF-7 cells were treated with MV (MOI 0.1) and/or (**B**) 10 nM, (**C**) 30 nM, (**D**) 50 nM of CPT in different models (drug sensitization, viral sensitization, and co-treatment), following which cell viability was determined by MTT assay. (**E**) The CI value of combination models were measured by Chou-Talalay method where CI value quantitatively defines synergism (CI < 1), additive effect (CI = 1) and antagonism (CI > 1). All data shown are means ± SEM (*P < 0.05 compared to MV treatment, and ^#^*P* < 0.05 compared to CPT treatment) from three independent experiments.

In order to explore the effects of combination therapy with oncolytic MV and CPT, we examined three different modes of treatment (schematically represented in Fig. 2A). These consisted of ‘drug sensitization’ (pre-treatment of cells with CPT prior to MV infection), ‘viral sensitization’ (pre-infection first with MV, followed by CPT treatment), and ‘co-treatment’ (concurrent addition of oncolytic MV and CPT) to determine the most effective treatment regimen. The data from each combination model were assessed by the Chou-Talalay method^28^ for level of synergy, where the CI value quantitatively defines synergism (CI < 1), additive effect (CI = 1), and antagonism (CI > 1). CPT was administered at 10 nM (Fig. 2B), 30 nM (Fig. 2C), or 50 nM (Fig. 2D) in combination with MV (MOI 0.1) and cytotoxicity was measured by MTT assay. Our results showed that the co-treatment model yielded the greatest cytotoxicity against MCF-7 cells, at all three concentrations (10, 30, and 50 nM) of CPT tested. This was evidenced by the reduction in MCF-7 cell viability to 44.45%, 34.87%, and 30.54% by MV plus 10, 30, or 50 nM CPT co-treatment, respectively (Fig. 2B–D). The percentage of cell death with co-treatment was 20 ~ 30% higher as compared to either MV or CPT treatment alone. Similar results were obtained using the LDH release detection assay (Supplementary Fig. S2A), and enhanced cytotoxic effect was noted by microscopy (Supplementary Fig. S2B). Consistently, the lowest CI values (0.11, 0.21, and 0.31) were obtained for 10, 30, and 50 nM CPT co-treatment (Fig. 2E), respectively, suggesting that the MV and CPT co-treatment model exhibits the strongest synergistic oncolytic activity against MCF-7 cells. On the other hand, in both drug sensitization and viral sensitization models, the CI values reflected antagonism, regardless of the CPT concentrations being tested (Fig. 2B–E).

### CPT treatment does not influence the early viral stages of oncolytic MV nor exert antiviral activity against MV infection

**Figure 3:**
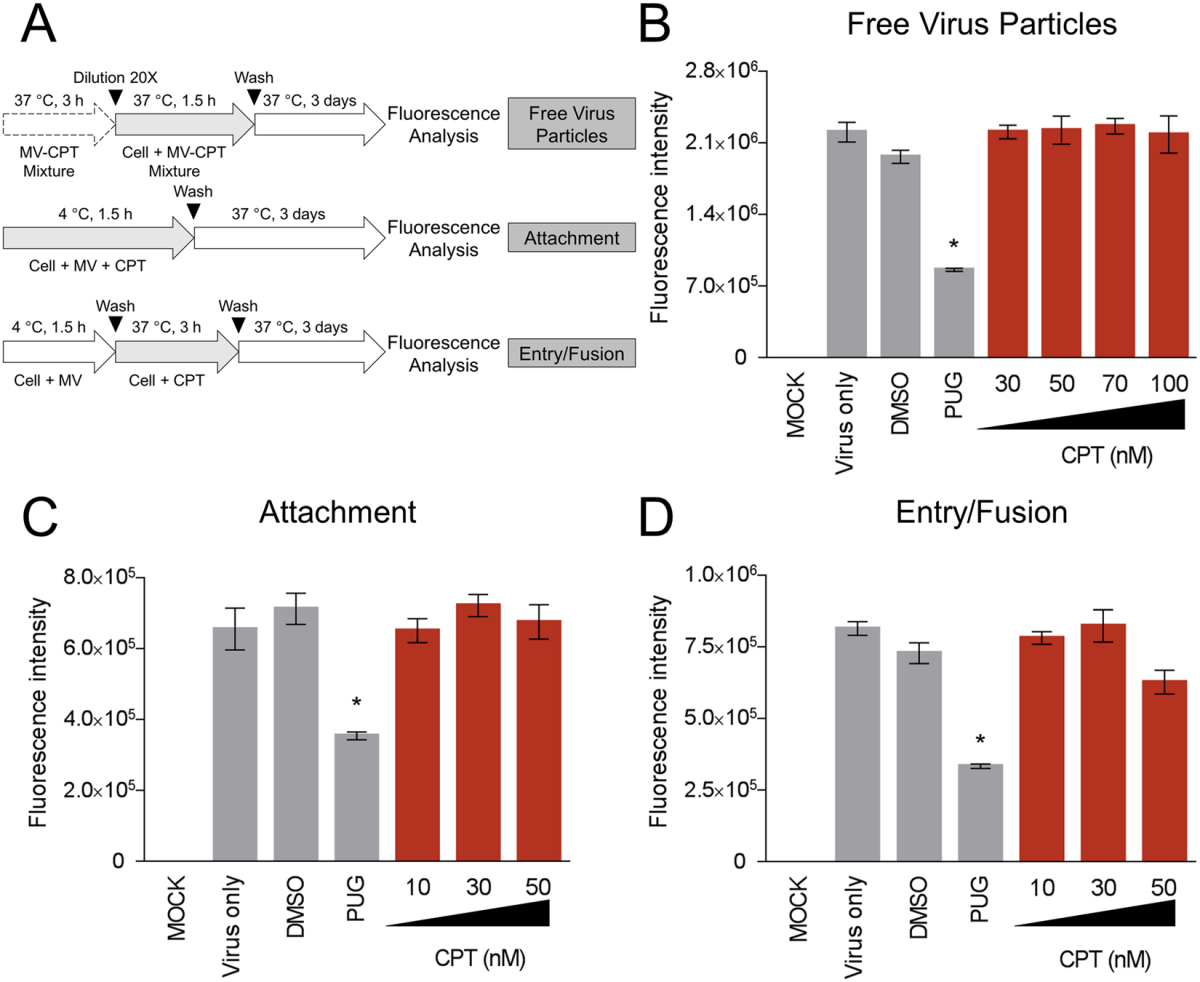
CPT treatment does not influence the viral entry steps of the oncolytic MV. (**A**) Schematic representations of synchronized infection analysis on early viral entry. (**B**) CPT’s effect on free MV particles. (**C**) CPT’s effect on MV attachment. (**D**) CPT’s effect on MV entry/fusion. For all experiments, final MV MOI was 0.1, DMSO (0.1%) was included as negative control, and PUG = 50 µM was included as positive control treatment in each condition. All data shown are means ± SEM (*P < 0.05 compared to Virus Only treatment) from three independent experiments.

**Figure 4:**
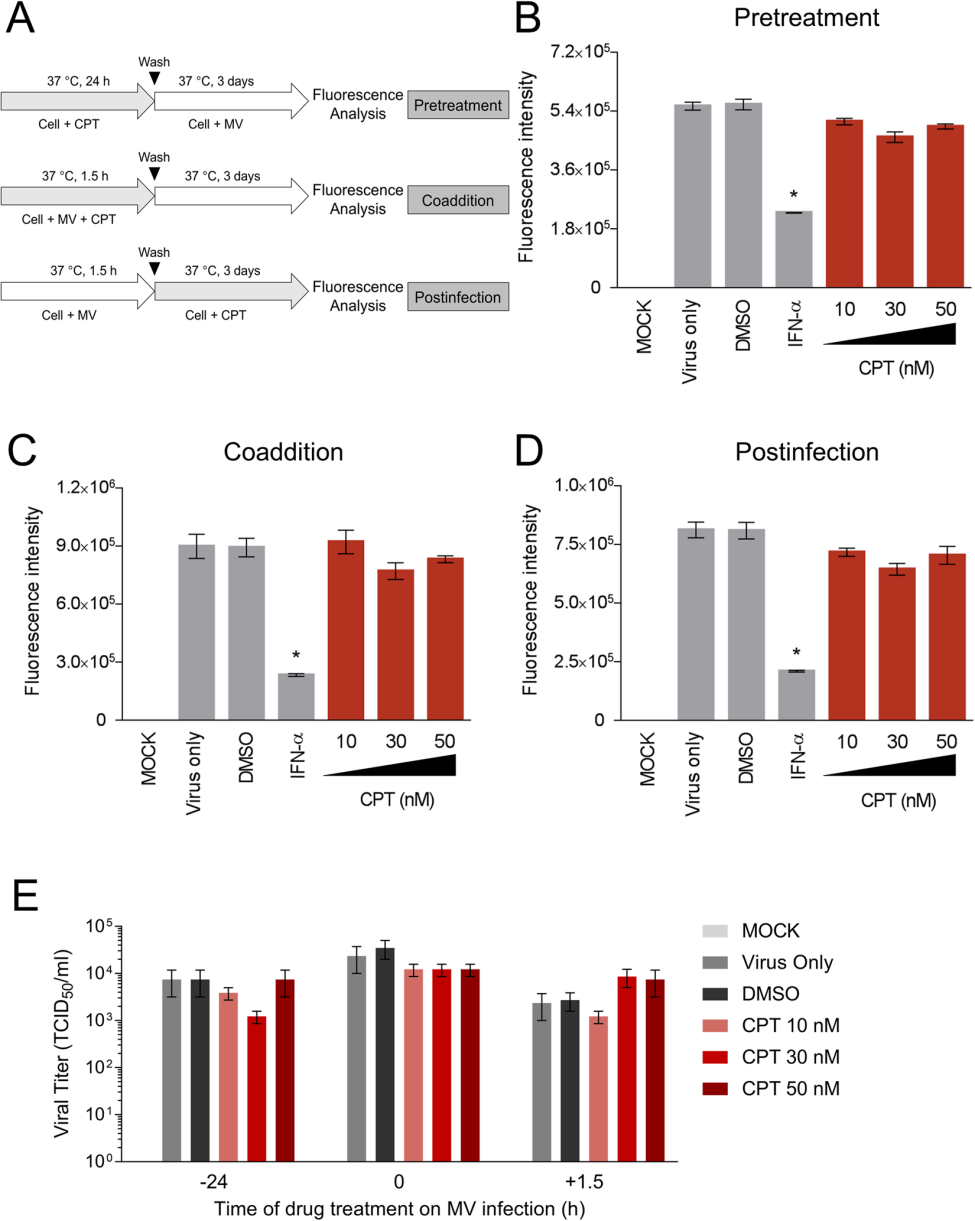
CPT treatment neither enhances nor exerts antiviral activity against MV infection. (**A**) Schematic representations of the time-of-drug-addition analysis on CPT treatment against MV infection. (**B**) Pre-treatment effect of CPT on MCF-7 cells before MV infection. (**C**) Co-addition treatment effect of CPT on MV infection of MCF-7 cells. (**D**) Post-infection treatment effect of CPT on MCF-7 cells immediately after MV infection. Results were obtained after 3 days of incubation. (**E**) Viral titer readouts of CPT treatment at different time-points of MV infection as in (**B**–**D**). For all experiments, MV infection was performed at MOI 0.1, DMSO (0.1%) was included as negative control, and IFN-α (1,000 IU/ml) was included as positive control treatment where indicated. All data shown are means ± SEM (*P < 0.05) from three independent experiments.

In order to examine potential antagonistic interaction(s) between co-administered CPT and MV, we first investigated whether CPT interferes with the early viral entry steps of MV infection of MCF-7 breast cancer cells. More precisely, we assessed the impact of CPT treatment on free oncolytic MV particles, its attachment to the host cells, and the post-binding viral entry/fusion steps during penetration of the cell (Fig. 3A). CPT was added to the virus and/or cells at specific time-points and viral EGFP reporter fluorescence was measured following incubation with the cells. As shown in Fig. 3B–D, CPT treatments at all doses tested did not produce significant inhibition during the early stages of virus infection. On the other hand, the positive control, punicalagin (PUG), a small molecule known to block MV entry stages^26^, effectively reduced MV infectivity. These results suggest that CPT does not inactivate cell-free MV particles or affect the early entry steps of MV infection in breast cancer cells. To rule out other potential antiviral effects, we used CPT to treat MCF-7 cells prior, during, and after infection with the oncolytic MV (Fig. 4A). Interferon-α (IFN-α) was included as control. As depicted in Fig. 4, pre-treatment of the breast cancer cells with CPT (Fig. 4B), concurrent addition of CPT to the MV inoculation (Fig. 4C), and post-infection CPT treatment (Fig. 4D) had minimal impact on the MV infectivity. Likewise, CPT treatment at any time-point from 24 h prior to and immediately after the MV infection did not cause significant difference on the resulting viral titers at the end-point of the experiment (Fig. 4E). These observations indicate that CPT, administered at the doses used in our experiments, does not elicit antiviral activity or perturb infections by oncolytic MV.

### Oncolytic MV and CPT combinatorial treatment causes sub-G1 cell cycle arrest and induction of apoptosis in human MCF-7 breast cancer cells

**Figure 5:**
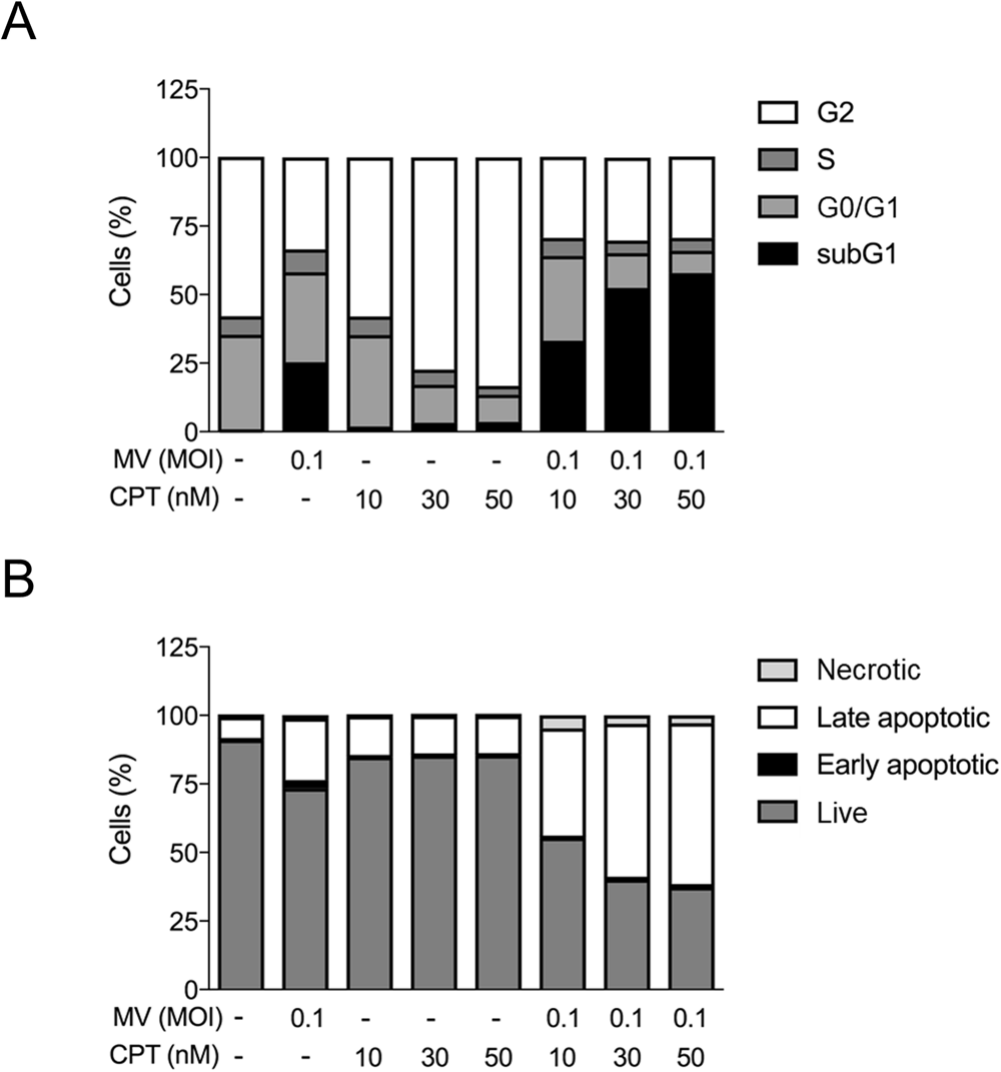
Oncolytic MV and CPT combinatorial treatment causes cell cycle arrest and induction of apoptosis in human MCF-7 breast cancer cells. (**A**) Flow cytometric cell cycle assay was performed using propidium iodide (PI) staining on MCF-7 cells following 5 days of treatment with CPT and MV in co-treatment model. Percentage of cells in sub G1, G0/G1, S or G2 phase were calculated by Beckman Cytomics FC500 Flow Cytometry CXP analysis. (**B**) Flow cytometric double stain assay was performed using propidium iodide (PI) and Annexin V (APC conjugated) staining on MCF-7 cells following 5 days of treatment with CPT and MV in co-treatment model. Percentage of cells that were necrotic, early apoptotic, late necrotic, or live was determined by Beckman Cytomics FC500 Flow Cytometry CXP analysis. Data shown are means ± SEM from three independent experiments.

**Figure 6:**
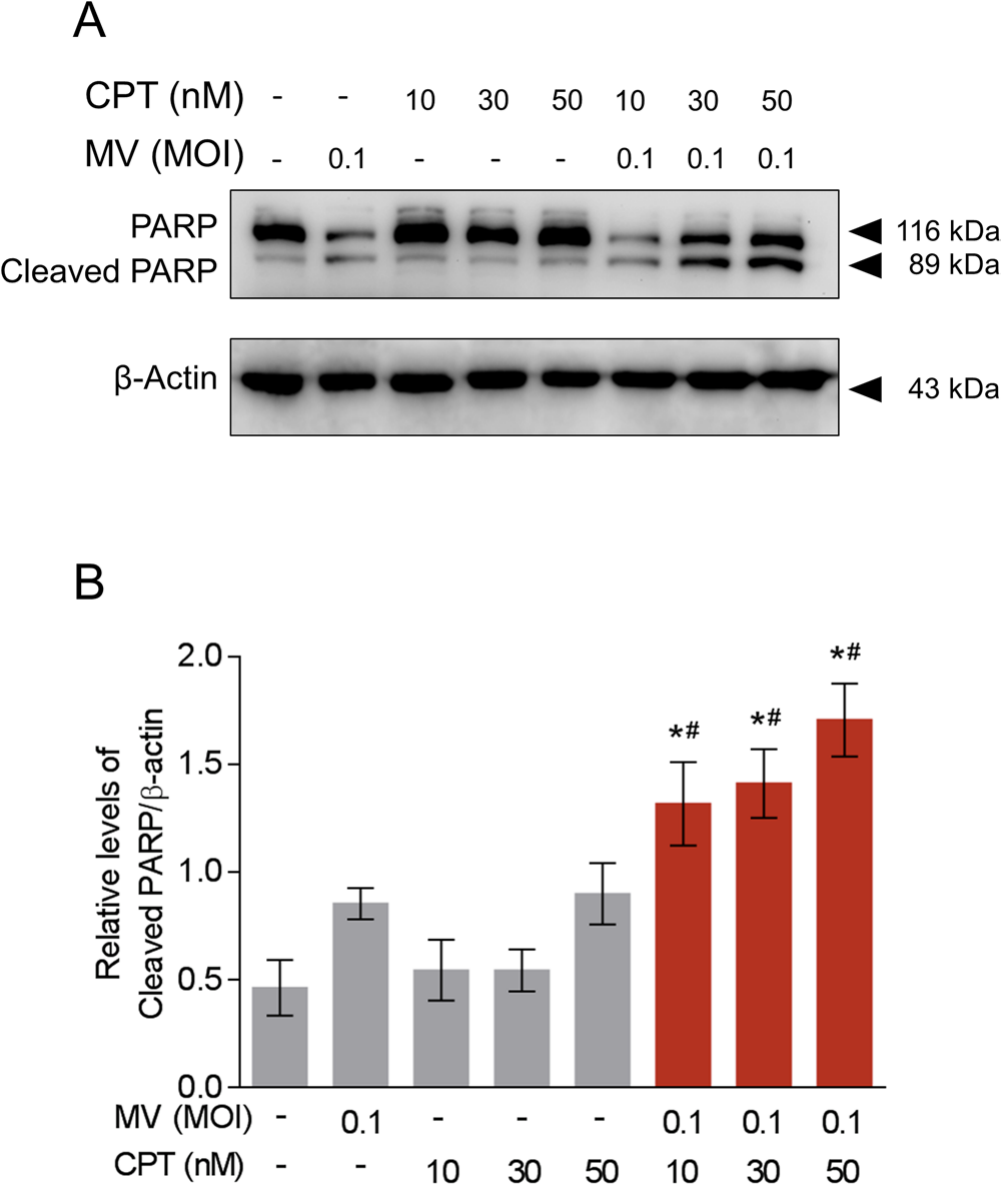
Combinatorial treatment of oncolytic MV with CPT induces apoptosis via PARP cleavage. (**A**) Western blot analysis of PARP expression from MCF-7 cells co-treated with CPT and MV for 5 days. (**B**) Quantitation of the level of cleaved PARP from (**A**). The representative Western blot and average quantitative data (means ± SEM) shown are from three independent experiments (*P < 0.05 compared to MV MOI 0.1 and ^#^*P* < 0.05 compared to CPT only at the same concentration).

To explore the underlying mechanism of the synergistic oncolytic effect elicited by co-treatment with MV plus CPT, we analyzed how the MCF-7 cell cycle was affected by the 2 agents using flow cytometry. Our results indicated that the oncolytic MV alone increased the sub-G1 cellular population to 25% as compared to untreated cells, while CPT alone dose-dependently induced G2/M cell cycle arrest at the three concentrations (10, 30, and 50 nM) tested (Fig. 5A). In contrast, as compared to treatment with each agent alone, the combination of MV and CPT treatment substantially increased the sub-G1 population at the three CPT doses used (10, 30, and 50 nM), together with corresponding reductions in G1 and G2 populations (Fig. 5A). The observed increase in sub-G1 population suggested that there was an increase in apoptotic cells. This premise was substantiated by flow cytometry using the Annexin V/PI double staining assay. We observed a shift in the number of apoptotic cells from about 25% with MV alone and 13% with CPT alone to 40% ~ 60% when both agents were used in combination (Fig. 5B). Finally, to further confirm that apoptosis was enhanced by co-treatment of MCF7 cells with MV and CPT, we analyzed PARP cleavage using Western blot analysis. Consistent with the Annexin V/PI analysis, levels of cleaved PARP, which are also indicative of apoptosis, were found to substantially increase in MCF-7 cells that were treated with a combination of MV and CPT (Fig. 6A). A dose-dependent increase in PARP cleavage was observed over the three different CPT concentrations (10, 30, and 50 nM) following densitometry analysis of the cleaved products (Fig. 6B). Taken together, our results suggest that the combination treatment consisting of oncolytic MV with CPT produces a synergistic oncolytic effect against human breast cancer cells, and this enhanced efficacy is mediated by increased levels of apoptosis.

### Combination of oncolytic MV and CPT exerts similar enhanced killing effect on T-47D breast cancer cells

**Figure 7:**
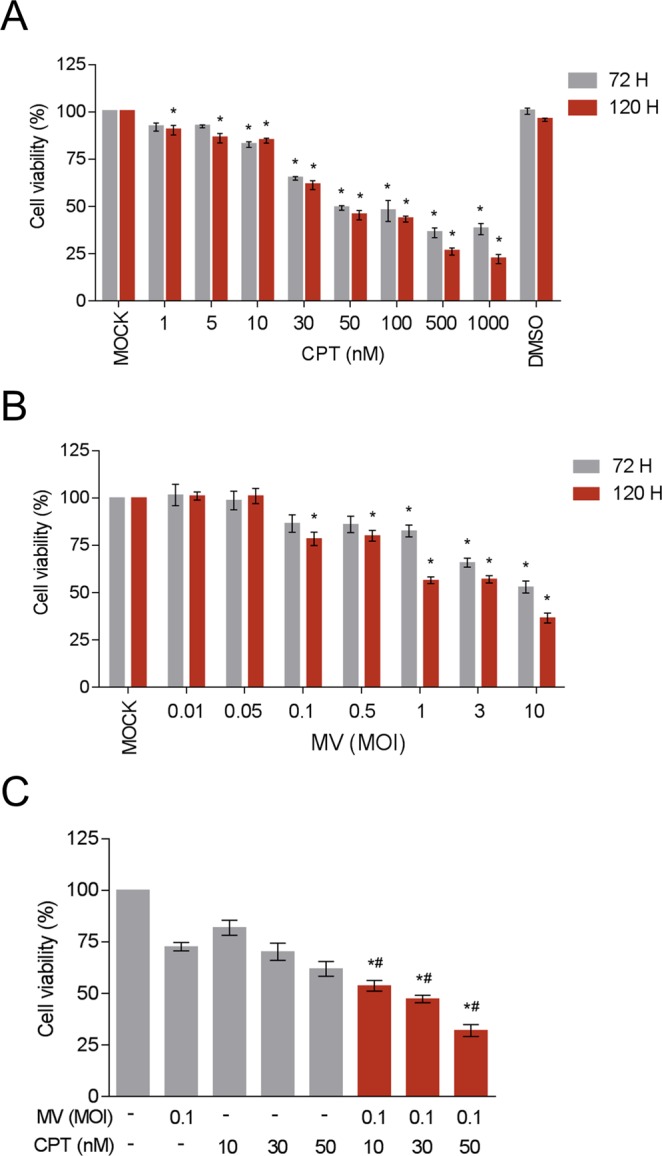
CPT and oncolytic MV are cytotoxic against human T-47D breast cancer cells and induce enhanced cell death in combination using the co-treatment model. T-47D cells (10^4^ cells per well) were treated with CPT (1–1000 nM) (**A**) and/or infected with MV (MOI 0.01, 0.05, 0.1, 0.5, 1, 3, and 10) (**B**) for 3 or 5 days. Cell viability was analyzed by MTT assay; DMSO = 0.1%. Data shown are means ± SEM (*P < 0.05 compared to Mock treatment) from three independent experiments. In the co-treatment model (C), T-47D cells were treated with MV (MOI 0.1) and CPT (10, 30, and 50 nM). Data shown are means ± SEM (*P < 0.05 compared to MV treatment, and ^#^*P* < 0.05 compared to CPT treatment) from three independent experiments.

Finally, to examine whether the combined treatment of oncolytic MV and CPT was effective against another breast cancer cell line, we also tested their influence as separate agents or in combination on the human breast cancer T-47D cells using the same methods and co-treatment model described in Figs 1 and 2, respectively. The T-47D cells are permissive to MV infection^12^, and were observed to be sensitive to a dose-dependent cytotoxic effect induced by CPT (10–50 nM) and MV (MOI 0.01–10) as separate agents, with 50 nM CPT and MV at MOI of 0.1 being near or below the 50% cell viability threshold (Fig. 7A,B). More importantly, the combined treatment of CPT (10, 30, and 50 nM) and MV (MOI 0.1) using the co-treatment model generated significantly more cell death compared to each agent alone (Fig. 7C). Comparable results were obtained between the MTT analysis (Fig. 7) and the LDH release detection assay (Supplementary Fig. S3).

Altogether, our results demonstrated that CPT and oncolytic MV can be used in combination using a co-treatment approach, and that the combined treatment induces enhanced cell death in the breast cancer cells.

## Discussion

In the current management of breast cancer, chemotherapy plays a major role in treating patients with larger tumor burdens, lymph node invasion, or recurrent/metastatic breast cancer^30^. The most commonly used chemotherapeutics for treating breast cancer include anthracyclines and taxanes, which are characterized by adverse effects such as cardiotoxicity^31^, neurotoxicity, alopecia^32^, and bone marrow suppression^31,32^. Oncolytic virotherapy using MV is potentially an effective strategy specifically targets tumor cells, but can also facilitate the detection and elimination of micro- and macrometastases^3^.

To overcome the limitation of viral oncolytics as a monotherapy, the combination of oncolytic virus and chemotherapeutic agents has been explored. This approach was explored based on the premise that the two treatment modalities may enhance or synergize with each other^33,34^, improving the outcome of cancer treatment. However, compatibility and synergism of the two types of treatment could be context-dependent^35^. To date, very few chemotherapeutics have been investigated in combination with MV therapy besides cyclophosphamide (CPA)^36,37^ and doxorubicin^38^. Our finding that co-treatment of CPT can boost MV efficacy adds to the list of therapeutic strategies that enhance MV oncolytics. Some other studies had explored the use of prodrug convertase-encoding MV to catalyze the conversion of chemotherapeutic prodrugs^15^. These include arming MV with purine nucleoside phosphorylase (MV-PNP)^39–42^, which converts fludarabine and 6-methylpurine-2′-deoxyriboside (MeP-dR) into 2-fluoroadenine and 6-methylpurine, respectively, or super cytosine deaminase (MV-SCD)^43–47^ that converts 5-fluorocytosine (5-FC) into 5-fluorouracil (5-FU). In the case of CPT, its clinically available analog irinotecan serves as a prodrug and is converted in the liver into the active metabolite and topoisomerase I inhibitor 7-ethyl-10-hydroxycamptothecin (SN-38), which circulate to kill the cancerous cells^48^. Eventually, the majority of SN-38 is transformed to its inactive metabolite SN-38 glucuronide (SN-38G) and released into the intestinal lumen for elimination, where bacterial beta-glucuronidase could regenerate SN-38 and results in the side effect of diarrhea^49^. Huang *et al*. has demonstrated that the combination of intratumoral injection of adenovirus expressing beta-glucuronidase and intravenous injection of irinotecan significantly enhanced the *in vivo* antitumor activity as compared to single-agent treatment^50^. This might indicate the potential use of beta-glucuronidase-expressing MV vector in combination with CPT or its derivatives as future strategies.

Given the limited expression of Nectin-4/PVRL4 in normal tissues including the skin, hair follicles, trachea, and lung^51,52^, but elevated expression in many adenocarcinomas such as breast, lung, bladder, pancreatic, and ovarian cancers^8,10,11,53,54^, Nectin-4/PVRL4 has emerged as an important tumor marker and therapeutic target. In breast cancer, it is a hallmark of advanced stage or highly metastatic cancer^11,55^ and promotes cell survival and proliferation by stimulating the c-Src kinase pathway^56^. In addition, a soluble form of Nectin-4/PVRL4 is present in the sera of breast and lung cancer patients^8,55^, which could have diagnostic applications for the screening of these cancers. Nectin-4/PVRL4 has also been proposed as a therapeutic target of primary and metastatic triple-negative breast cancers, as well as of lung, bladder, and pancreatic cancers which could potentially be treated with Nectin-4/PVRL4 antibodies conjugated to anti-neoplastic agents^57,58^. Compared to vaccine strain, the wild-type MV is more specific to Nectin-4/PVRL4 since it does not engage CD46^14^. The above reasons along with the results from our study suggest that wild-type MV backbone may serve as a suitable oncolytic vector for treating breast cancer.

In this study, we demonstrated, for the first time, that recombinant wild-type MV combined with low doses of CPT (10, 30, or 50 nM) enhances oncolytic killing of human breast cancer cells. We illustrated a synergistic killing effect during the co-treatment of these cells with both agents. Mechanistically, the synergistic combination treatment increased the accumulation of sub-G1 cell population and led to enhanced apoptosis as evidenced by elevated levels of cleaved PARP (Figs 5 and 6). Given that both MV and CPT treatments can each eventually lead to the induction of cellular apoptosis^21–24^, this outcome was largely anticipated. Interestingly, MV infection is known to induce autophagy as a pro-viral mechanism, wherein sustained autophagy delays apoptosis and facilitates MV cell-to-cell transmission or syncytia formation before the eventual cell death^59^. On the other hand, CPT has been observed to induce both autophagy and apoptosis^60^, with low-doses (50 nM and less) being capable of triggering premature senescence and autophagy^61^. Since both MV and CPT at the concentrations used in this study are known to induce autophagy, co-treatment of CPT and oncolytic MV could potentially amplify the autophagy process, leading to a better viral spread and further sensitizing the breast cancer cells to the eventual apoptotic cell death. Indeed, preliminary experiment indicates induction of the autophagy marker LC3 (‘LC3II’) with monotreatments using CPT or MV at 24 h and 48 h post-addition, respectively (Supplementary Fig. S4A). Interestingly, at 48 h post-treatment, we noted a concomitant decrease in the lipidation of LC3II with increasing CPT concentration in combination with MV (Supplementary Fig. S4A). This observation was likely not due to inhibition of autophagy but rather its potentiation (“faster turnover”), since treatment with the lysosomal inhibitor bafilomycin to block the autophagic flux caused a substantial change in the accumulation of LC3II in the CPT treatment groups with and without MV combination, as compared to MV infection alone (Supplementary Fig. S4B). Such amplification by CPT treatment on the inefficient autophagic flux induced by MV could potentially lead to autophagic flux perturbation, an event previously observed to promote apoptotic cell death^62^. Further experiments are required to explore this phenomenon and fully elucidate the nuance of virus- and drug-induced autophagy, prior to the cells undergoing apoptosis in the observed synergistic effect from MV and CPT combination.

Altogether, our results suggest that the oncolytic MV plus CPT chemovirotherapy is a potential synergistic combination treatment against breast cancer cells. CPT and MV act together since CPT does not have an antiviral effect when used alone. The synergistic therapeutic effects of the combinatorial treatment also reduce the effective dosages required for each agent. Our findings demonstrated that low doses of CPT (10, 30, and 50 nM) combined with lower MOI of MV (MOI 0.1) gave similar therapeutic effects as high doses of CPT (100 nM) and high MOI of MV (MOI 3) alone in breast cancer cells. This synergistic effect could lower toxicity associated with each reagent^25^, particularly the bone marrow suppression and gastrointestinal toxicity that has been reported for CPT and its derivatives topotecan^63^ and irinotecan^64^. In conclusion, the data presented in this paper emphasizes the importance of combination chemovirotherapy and support the future development of co-treatment with oncolytic MV and CPT as a potential therapy for the management of breast cancer.

## Supplementary information

SUPPLEMENTARY INFO
