# Supplementary material for: Chemovirotherapeutic Treatment Using Camptothecin Enhances Oncolytic Measles Virus-Mediated Killing of Breast Cancer Cells
Source: Sci Rep. 2019 May 1;9:6767. doi: 10.1038/s41598-019-43047-3 (PMC6494908; doi:10.1038/s41598-019-43047-3)

Supplementary Information

Chemovirotherapeutic Treatment Using Camptothecin Enhances Oncolytic Measles Virus-Mediated Killing of Breast Cancer Cells

Chen-Jei Tai[1,2,#], Ching-Hsuan Liu[3,4,#], Yu-Chi Pan[5], Shu Hui Wong[6], Cheng-Jeng Tai[7,8], Christopher D. Richardson[4,9] & Liang-Tzung Lin[3,5,*]

[1] Department of Traditional Chinese Medicine, Taipei Medical University Hospital, Taipei, Taiwan

[2] Department of Obstetrics and Gynecology, School of Medicine, College of Medicine, Taipei Medical University, Taipei, Taiwan

[3] Graduate Institute of Medical Sciences, College of Medicine, Taipei Medical University, Taipei, Taiwan

[4] Department of Microbiology and Immunology, Dalhousie University, Halifax, Nova Scotia, Canada

[5] Department of Microbiology and Immunology, School of Medicine, College of Medicine, Taipei Medical University, Taipei, Taiwan

[6] International Master Program in Medicine, College of Medicine, Taipei Medical University, Taipei, Taiwan

[7] Division of Hematology and Oncology, Department of Internal Medicine, Taipei Medical University Hospital, Taipei, Taiwan

[8] Department of Internal Medicine, School of Medicine, College of Medicine, Taipei Medical University, Taipei, Taiwan

[9] Department of Pediatrics and Canadian Center for Vaccinology, Izaak Walton Killam Health Centre, Halifax, Nova Scotia, Canada

[#]These authors share first authorship.

***CORRESPONDENCE**

Liang-Tzung Lin: Department of Microbiology and Immunology, School of Medicine, College of Medicine, Taipei Medical University, No. 250, Wu-Hsing Street, Taipei 11031, Taiwan, Tel: +886-2-2736-1661 ext. 3911, Fax: +886-2-2736-1661 ext. 3921, E-mail: ltlin@tmu.edu.tw.

Supplementary Figure S1. Cytotoxicity of CPT and oncolytic MV on human MCF-7 breast cancer cells determined by LDH release assay.

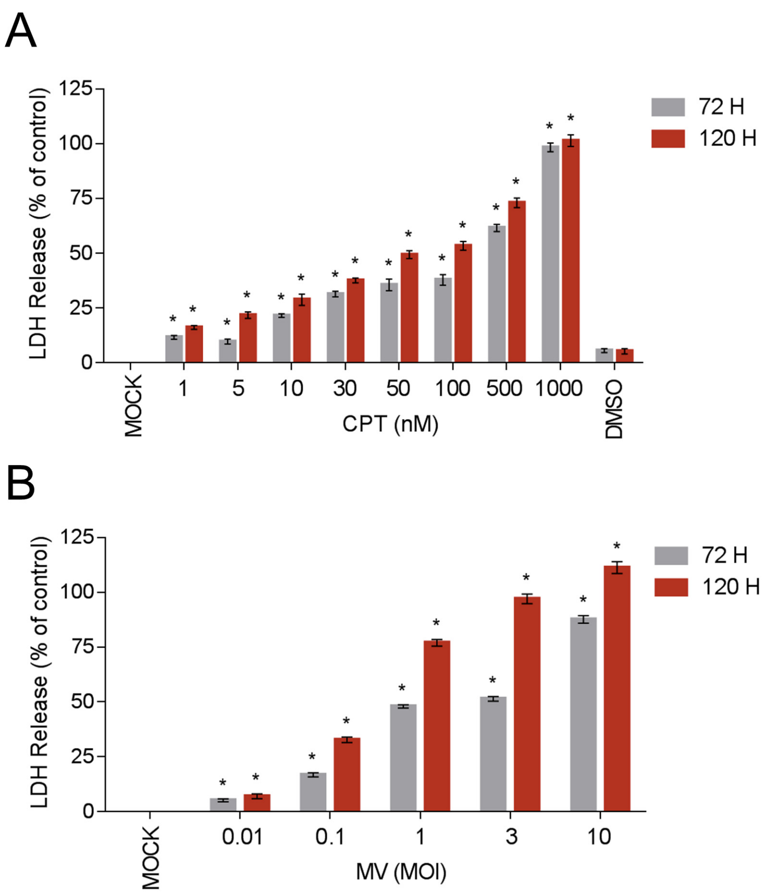

MCF-7 cells (10^4 cells per well) were treated with CPT (1-1000 nM) (A) or infected with MV (MOI 0.01, 0.1, 1, 3, and 10) (B) for 3 or 5 days and analyzed with lactate dehydrogenase (LDH) cytotoxicity detection kit (Takara Bio Inc.; Kusatsu, Japan). Following the manufacturer's instructions, supernatant from each well was transferred to 96-well plates, mixed with reaction substrates, and incubated at 37℃ for 30 min before the absorbance was measured at 490 nm using a microplate reader. DMSO = 0.1%. All data shown are means ± SEM (*$P < 0.05$ compared to Mock treatment) from three independent experiments.

Supplementary Figure S2. Effect of MV and CPT co-treatment on human MCF-7 breast cancer cells.

A

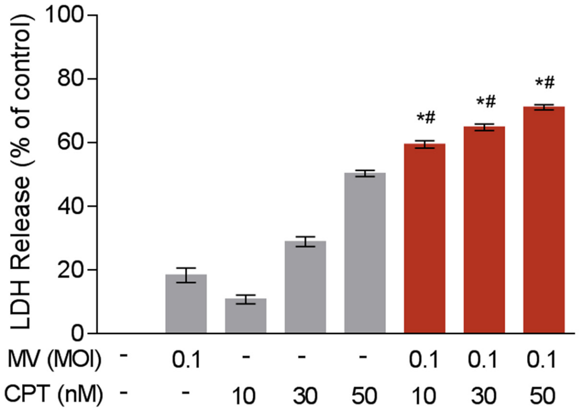

B

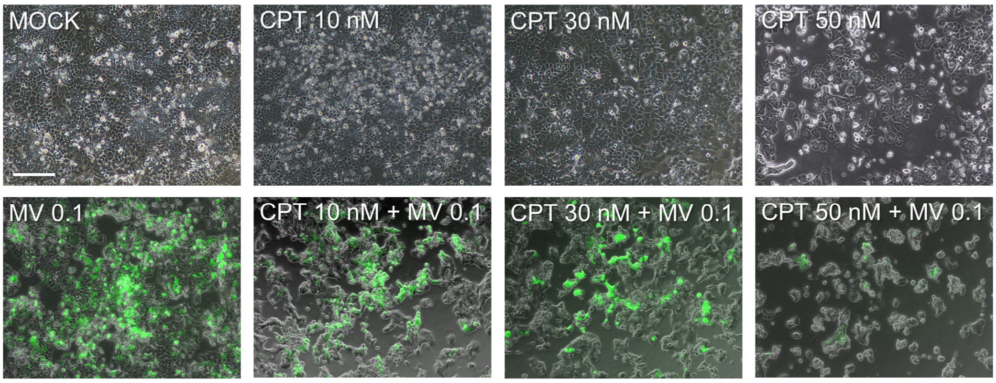

MCF-7 cells were treated with MV (MOI 0.1) and/or CPT (10, 30, and 50 nM) using the co-treatment model described in the text. Cell viability was then determined by LDH release assay (A) as described in Figure S1, and cell morphology was observed by microscopy (B). Data shown in (A) are means ± SEM (**P* < 0.05 compared to MV treatment, and $^{\#}P < 0.05$ compared to CPT treatment) from three independent experiments. Representative micrographs (B) are displayed in overlaid images of bright field (phase) with the corresponding dark field (fluorescence) pictures. Scale bar = 100 μm.

Supplementary Figure S3. Cytotoxicity of CPT and oncolytic MV on human T-47D breast cancer cells determined by LDH release assay.

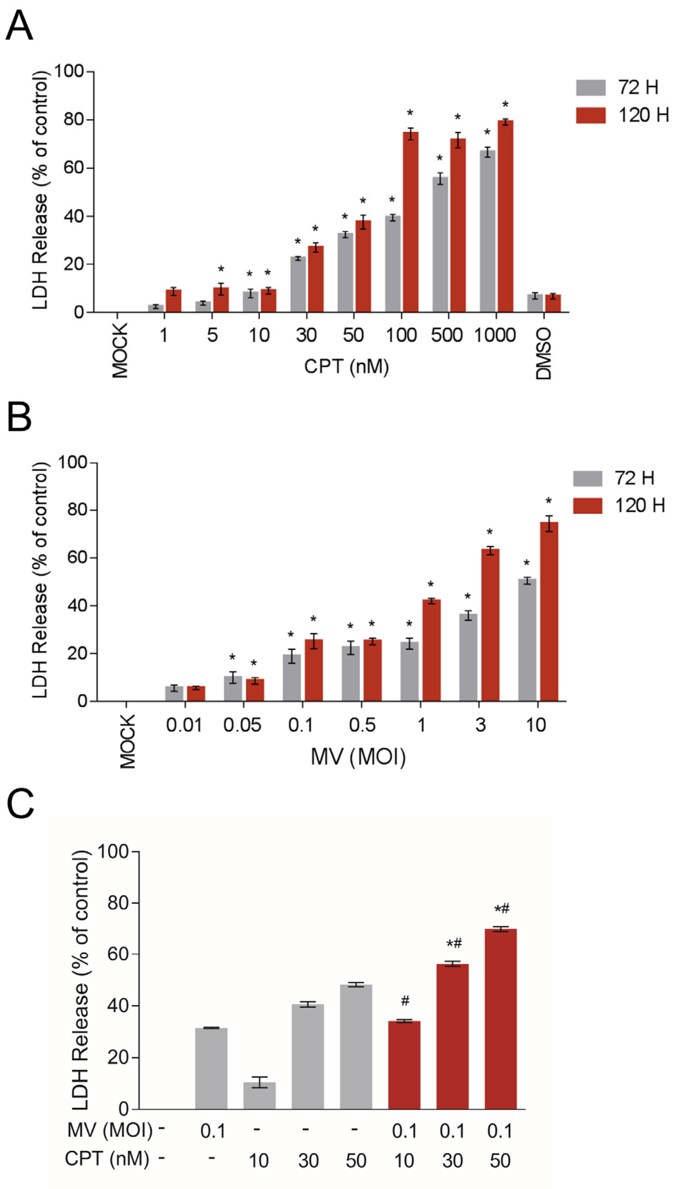

T-47D cells (10^4 cells per well) were treated with CPT (1-1000 nM) (A) and/or infected with MV (MOI 0.01, 0.05, 0.1, 0.5, 1, 3, and 10) (B) for 3 or 5 days before analysis by LDH release assay as described in Figure S1; DMSO = 0.1%. Data shown are mean ± SEM (*$P < 0.05$ compared to Mock treatment) from three independent experiments. In the co-treatment model (C), T-47D cells were treated with MV (MOI 0.1) and CPT (10, 30, and 50 nM) before LDH release was measured. Data shown are means ± SEM (*$P < 0.05$ compared to MV treatment, and $^{\#}P < 0.05$ compared to CPT treatment) from three independent experiments.

Supplementary Figure S4. Influence of MV and CPT co-treatment on autophagy.

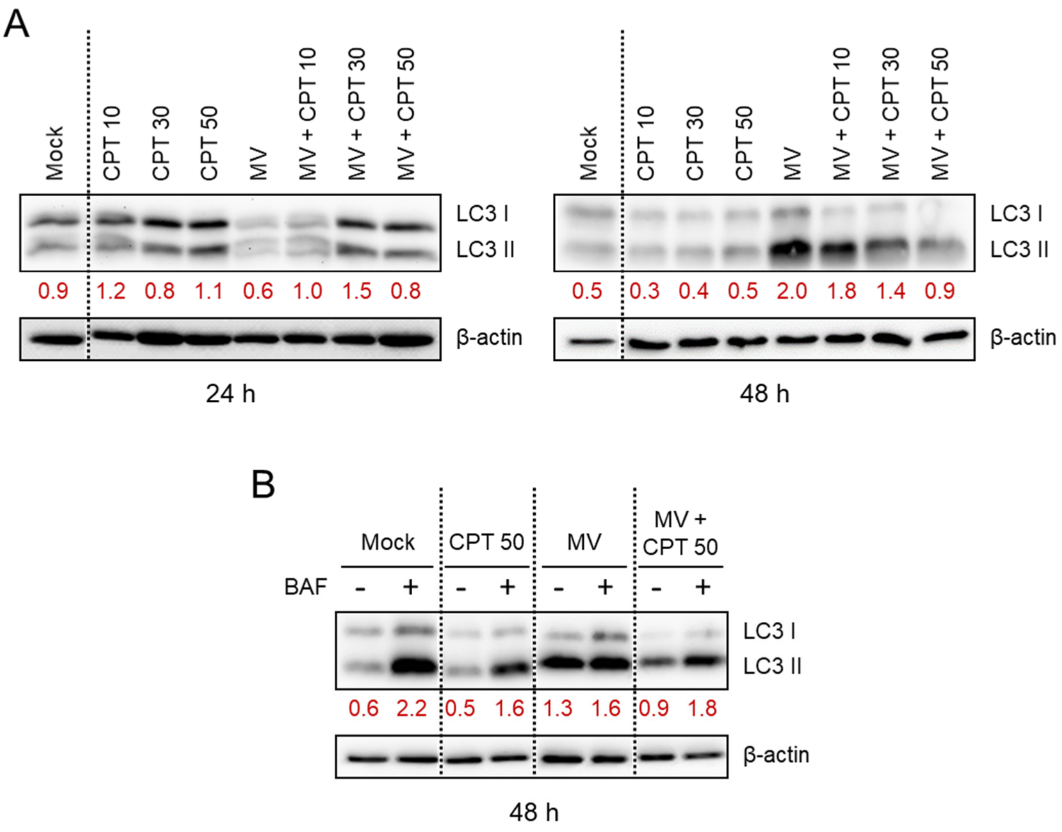

MCF-7 cells seeded in 12-well plates (2 x 10^5 cells per well) were treated with MV (MOI 0.1) and/or CPT (10, 30, and 50 nM) using the co-treatment model described in the text. (A) After 24 or 48 h, cells were harvested and analyzed with Western immunoblotting as described in the text. (B) Cells were treated with bafilomycin A1 (BAF; Sigma-Aldrich) 4 h before harvesting at 48 h. Antibodies were used to probe LC3 (1:1000; Thermo Fisher Scientific) and β-actin (1:10000; Cell Signaling Technology). LC3 II signals were quantified and compared against the β-actin loading control using densitometry analysis.

Supplementary Figure S5. Full-length Western blots of Figure 6A.

PARP and cleaved PARP:

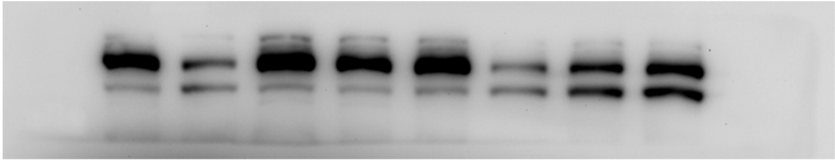

β-actin:

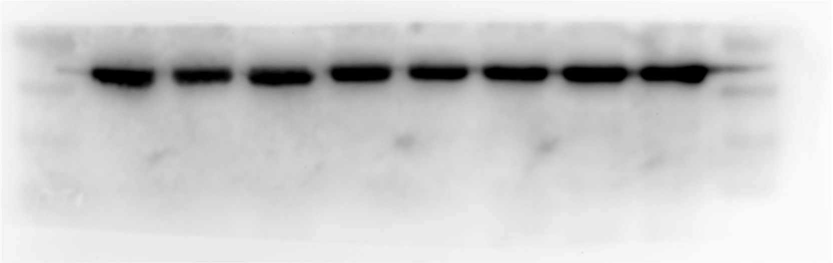

Supplement: Supplementary file 1 — SUPPLEMENTARY INFO [file 41598_2019_43047_MOESM1_ESM.pdf]
